# The impact of electroconvulsive therapy on the spatial QRS-T angle and cardiac troponin T concentration in psychiatric patients

**DOI:** 10.1371/journal.pone.0224020

**Published:** 2019-10-23

**Authors:** Michał Próchnicki, Grzegorz Rudzki, Michał Dzikowski, Andrzej Jaroszyński, Hanna Karakula-Juchnowicz

**Affiliations:** 1 I Department of Psychiatry, Psychotherapy and Early Intervention, Medical University of Lublin, Lublin, Poland; 2 Department of Endocrinology, Medical University of Lublin, Lublin, Poland; 3 Department of Family Medicine and Geriatrics, Jan Kochanowski University, Kielce, Poland; 4 Department of Clinical Neuropsychiatry, Medical University of Lublin, Lublin, Poland; University of Dundee, UNITED KINGDOM

## Abstract

**Background:**

Electroconvulsive therapy (ECT) is an effective treatment method used in psychiatry; however, its cardiac safety has not been clearly demonstrated. The aim of the study was evaluation of the ECT effects on the myocardium based on troponin T concentrations and the following ECG parameters: the spatial QRS-T angle (QRS-TA), QRS duration (QRSd) and the corrected QT interval (QTc).

**Methods:**

In the study 44 patients (12 female and 32 male) were enrolled diagnosed with schizophrenia (n = 21) and major depressive disorders (n = 23), according to the DSM-IVR criteria. All cases were undergoing ECT procedures. The mean age of the patients was 36.9±16 years (range: 18–74). Resting ECG was recorded before performing ECG and 1 hour after. The spatial QRS-TA was reconstructed from 12-lead ECG using the inverse Dower method. Troponin T concentration was assessed before the procedure and 6 hours after ECT.

**Results:**

No significant changes to troponin T concentrations were observed during the ECT series. The pre-ECT value of the spatial QRS-TA was 41.1±18.9°. The follow-up examinations did not reveal any significant increase of this parameter (p = 0.09) in any of the consecutive measurements. There were no significant changes in the QTc interval duration or the QRS complex duration demonstrated before the third, fifth and last procedure in the cycle (p>0.05). No significant changes to troponin T concentrations were observed during the ECT series.

**Conclusions:**

Our findings indicate a lack of negative ECT effects on the risk of adverse cardiovascular events measured by the spatial QRS-T angle and cardiac troponin T concentration.

## Introduction

Electroconvulsive therapy (ECT) is an effective treatment method in psychiatry, predominantly used to treat patients with severe,drug-resistant mental disorders, as well as to monitor acute, life-threatening symptoms of depression, mania and psychosis [1]. ECT is considered to be a relatively safe method [2]. However, the ECT-associated mortality is estimated to be 1/1000–1/10 000 patients or 1 death per 80 000 procedures [3]. One of the recent studies demonstrated elevated concentrations of cardiac troponins following ECT in several percent of patients [4]. Moreover, the literature reports acute ECT-related cardiovascular complications, such as myocardial infarction, ventricular arrhythmia, takotsubo cardiomyopathy, pulmonary embolism, and cardiac arrest in psychiatric patients[5–8].

Therefore, further studies regarding the ECT cardiac safety and risk stratification are needed. Cardiac troponins are considered to be useful and sensitive markers of cardiomyocyte structural damage, regardless of the baseline cause and other negative prognostic factors [9]. Routine electrocardiography (ECG) and other advanced techniques, such as digital analysis and the evaluation of the spatial QRS-T angle (QRS-TA), can be useful, easy-to-use examinations simplifying cardiovascular risk stratification [10]. We decided to use the spatial QRS-TA because the research findings indicated its high sensitivity as a predictive factor of cardiovascular complications. In this study the spatial QRS-TA was defined as the angle between the maximal QRS- and T-wave vectors in three-dimensional space, which reflects a deviation between ventricular depolarization and repolarization, and cardiac action potential inhomogenities. It has been demonstrated that the spatial QRS-TA is a strong predictor of cardiovascular death, sudden cardiac death, and non-fatal cardiovascular incidents, and is superior to other electrocardiographic and clinical predictive factors [11, 12].

The aim of the study was to evaluate the ECT effects on the myocardium using cardiac troponin T (cTnT) concentrations and the following ECG parameters: the spatial QRS-TA, QRS duration (QRSd) and the corrected QT interval duration (QTc), analyzed in resting ECG performed before and after the procedure.

## Material and methods

### Ethics statement

Written informed consent of participation in the study was obtained from every patient (Approval of the Bioethics Committee of Medical University of Lublin No. KE-0254/77/2012). The study was conducted according to the Declaration of Helsinki principles. The patients’ capacity to consent, including their current mental condition and cognitive functions, was assessed by an independent psychiatrist before the study. None of the participating patients was legally incapacitated.

### Study population and setting

The patients were recruited at the 1^st^ and 2^nd^ Department of Psychiatry, Medical University of Lublin. The study involved 44 patients, including 12 women and 32 men, diagnosed with schizophrenia (n = 21) and depression (n = 23). The mean age of the patients was 36.9±16 years (a range of 18–74 years); 10 cases (22.7%) had been previously treated with ECT. The indications for ECT therapy were drug resistance, acute deterioration of mental state requiring immediate and effective therapy, and a history of good response to prior ECT courses [2]. The exclusion criterion was the lack of the patient’s cooperation, as well as conditions disrupting ECG analysis (i.e. intraventricular conduction delay, atrial fibrillation, artificial pacing).

Prior to ECT, routine, medical history was obtained and the patients underwent physical examinations performed by an independent physician, a neurologist and an anesthesiologist; all available medical records were analyzed. ECG and other laboratory tests necessary for qualification were performed. The concomitant diseases included arterial hypertension (n = 8), type 2 diabetes mellitus (n = 5), hypercholesterolemia (n = 15), obesity (n = 9) and nicotine dependence (n = 23) (Table 1).

**Table 1:** Comorbidities and medications in the study group.

	n (%)
Comorbidity	
Arterial hypertension	8 (18%)
Type 2 diabetes mellitus	5 (11%)
Hypercholesterolemia	15 (34%)
Obesity	9 (20%)
Medications	
ACE inhibitors/ AT_1_ receptor antagonists	6 (14%)
β receptor antagonists	4 (9%)
Diuretics	3 (7%)
Metformin	5 (11%)
Sulphonylourea derivates	2 (5%)
Statins	12 (27%)

All the patients were undergoing psycho- and pharmacotherapy, and were taking antidepressants and/or antipsychotic drugs. During the study, the patients were administered the following psychotropic drugs: tricyclic antidepressants (TCA): n = 3 (7.5%) (including amitriptyline n = 3); noradrenergic and specific serotonergic antidepressants (NaSSA): n = 13 (32.5%) (including: mianserin n = 8, mirtazapine n = 5); serotonin and norepinephrine reuptake inhibitors (SNRI): n = 5 (12.5%) (including venlafaxine n = 5); serotonin antagonists and reuptake inhibitors (SARI): n = 1 (2.5%) patients (including trazodone n = 1); selective serotonin reuptake inhibitors (SSRI): n = 9 (22.5%) including (fluoxetine n = 2, sertraline n = 4, escitalopram n = 3); antagonist activity at 5HT_2C_ receptors and agonist activity at melatonergic MT_1_/MT_2_ receptors: agomelatine n = 1 (2.5%), first-generation antipsychotics: n = 21 (52.5%) (including zuclopenthixol n = 1, haloperidol n = 6, perazine n = 8, flupentixol n = 1, perphenazine n = 1, levomepromazine n = 4, chlorprothixene n = 2); second-generation antipsychotic drugs n = 23 (57.5%) (including amisulpride n = 4, aripiprazole n = 3, clozapine n = 3, sulpiride n = 3, quetiapine n = 4, risperidone n = 2, olanzapine n = 10).

None of the patients was treated with benzodiazepines or normothymic drugs.

### Electroconvulsive therapy (ECT)

The patients underwent bilateral ECT procedures using the Thymatron System IV (Somatics, LLC) device at standard parameters as previously described [13]. The procedures were performed under intravenous anesthesia with propofol and suxamethonium, as well as atropine, in the appropriate doses [14, 15]. EEG monitoring was used to confirm the occurrence of seizures. All ECT procedures analyzed in this work were effective and induced ≥30-seconds seizures. The ECT cycle consisted of 9–12 procedures carried out twice a week. The analyses were performed before the onset of treatment (the 1st procedure), in the middle of therapy (the 5th procedure) and after the 9th procedure. Since only a few patients had more than nine ECT procedures, these interventions were not included in the final analysis.

### Electrocardiography

Resting ECG was recorded in the supine position of the patients, using the MAC 5500 device (GE Healthcare, USA) and the standard 12-lead system. ECG was recorded for 10 seconds before ECT and 1 hour after. The sampling frequency was 500 sps.

The digital form of ECG tracings were automatically averaged to a single beat and transformed into vectorcardiographic recordings. Transformation into three orthogonal leads X, Y and Z was carried out according to the inverse-Dower matrix. This approach allowed to obtain projections of the maximum vectors of QRS and T waves onto the frontal (xy), transverse (xz) and right sagittal (yz) planes, and onto axes x, y and z. Subsequently, the maximum spatial QRS vector, the maximum spatial T vector and the spatial QRS-T angle between them were calculated using the equations previously published [16]. Spatial QRST angle values > 50 degrees are considered abnormal. Previous study by Yamazaki et al. [12] demonstrated that QRS-TA was an independent risk factor for annual cardiovascular mortality (1.5- and 1.9-fold higher risk for QRS-TA 50–100 degrees and >100 degrees, respectively).

Heart rate, duration of the QRS complex (QRSd) and the corrected QT interval (QTc) were automatically calculated based on a 10-second 12-lead ECG recording. The Bazett’s formula was used to correct QT intervals of heart rate (QTc = QT/√RR) [17]. The values of QRSd >100 ms and the QTc increase of 60 ms from baseline were considered abnormal; normal values of QTc are below 440 ms in males, and 450 ms in females. However, a cut-off point of 500 ms is associated with a high risk of ventricular arrhythmia [18]. Furthermore, ST changes and T wave inversions were analyzed.

### Biochemical measurements

Serum concentrations of troponin T were determined before and 6 hours after the ECT procedures. The concentrations of troponin T were determined by the immunochemiluminescence assay using an automated analyzer Cobas 6000 E601 (Roche Diagnostics GmbH, Hitachi High Technologies Corporation, Japan). The detection threshold was 0.003 ng/ml, and the values above the 99th percentile, i.e. ≥0.01 ng/ml, were considered abnormal. In addition, serum potassium, sodium, creatinine thyroid stimulation hormone and complete blood cells count were measured in order to eliminate the impact on non-cardiac disturbances on ECG.

### Statistical analysis

STATISTICA 12 software (Statsoft Inc. 1984–2014, USA) was used for the statistical analysis and *p<0*.*05* was considered statistically significant in all calculations. The results were presented as the mean and standard deviations. The normality of distribution was evaluated using the Shapiro-Wilk W test. The analysis of variance (ANOVA) for repeated measurements was applied to determine the significance of differences between the consecutive sessions of ECT.

## Results

### Spatial QRS-T angle and ECG parameters

The pre-ECT value of spatial QRS-TA was 41.1±18.9° (Table 2). The follow-up examinations did not reveal any significant increase in the parameter (*p = 0*.*09*) in any of the consecutive measurements. The spatial QTS-TA did not exceed 100 degrees; its value was above 50 degrees only in two patients, both before and after the procedures (53 and 54 degrees in one patient; 59 and 64 degrees in second one).

**Table 2:** The effect of ECT on ECG parameters.

ECG parameter	1st ECT session		5th ECT session		9th ECT session		*F*	*p*
	before	after	before	after	before	after		
Spatial QRS-TA (°)	41.1±18.9	43.2±11.7	42.2±8.9	42.8±9.2	40.6±1.3	42.4±9	*1*.*54*	*0*.*09*
Spatial QRS-TA >50° (n)	2	2	2	2	2	2	*NA*	*NA*
QRSd (ms)	92.8±5.6	92.4±6.4	93.2±2.8	93.8±7.9	95.2±7.3	94.9±5.8	*1*.*87*	*0*.*74*
QTc (ms)	430.2±26.4	419.8±30.9	434.3±27.3	422.2±13.1	428.8±29.1	425.8±19.6	*1*.*54*	*0*.*11*
Heart rate (bpm)	82.1±4.5	80.4±5.1	79.9±4.1	82.4±4.9	84.2±7.3	78.2±4.6	*1*.*18*	*0*.*56*
ST/T abnormalities (n)	1	1	1	1	1	1	*NA*	*NA*

Data expressed as the mean ± SD. The analysis of variance (ANOVA) for repeated measurements was applied to determine the significance of differences between consecutive measurements of QRS-TA, QRSd, QTc.

Abbreviations: QRS-TA–QRS-T angle, QRSd–duration of QRS complex, QTc–corrected QT interval duration, NA–not applicable.

We found baseline ST segment depression in only one patient without cardiac disease. This abnormality was persistent throughout all the cycles of ECT. None of the patients presented ST-T abnormalities *de novo* while treated with ECT.

The pre-ECT QTc interval was 430.2±26.4 ms. There were no significant changes observed during the following cycles (*p = 0*.*11*) (Table 2). None of participants demonstrated with QTc >500 ms either at baseline or during consecutive cycles of therapy. A QTc increase of 60 ms from baseline was not found in any patient.

The duration of the QRS complex was 92.8±5.6 ms and did not significantly change during the cycle of ECT procedures (*p = 0*.*74*). At baseline five patients had QRSd 101–110 ms which persisted throughout the subsequent cycles.

There was no significant correlation between QRSd and QTc and QRS-TA. Patients with ST-T abnormalities (n = 1) and prolonged QRSd (n = 5) did not present with increase of QRS-TA when compared with whole study group.

### Cardiac troponin T concentration

**Fig 1:**
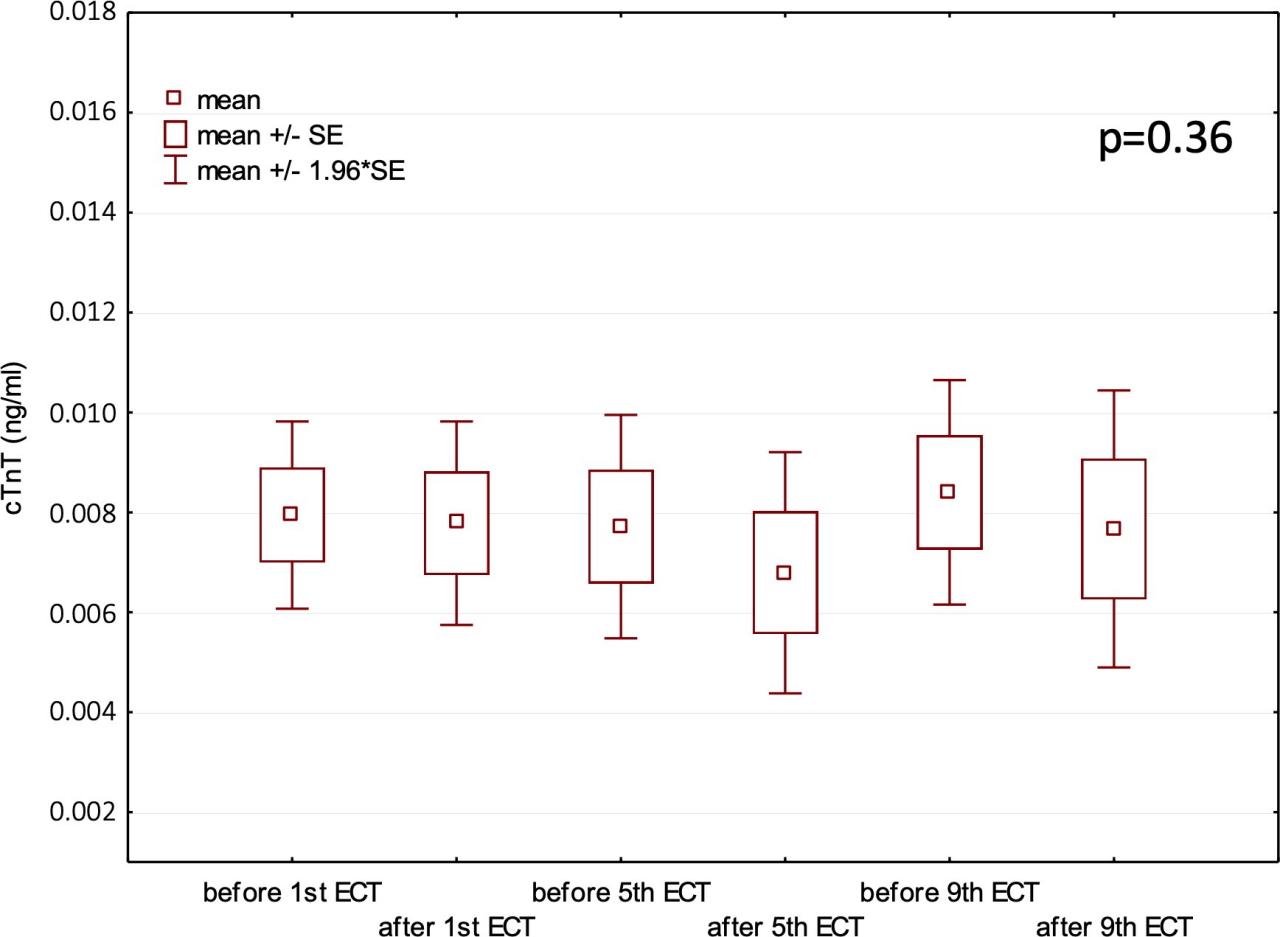
The effect of ECT on cTnT concentration.

We found no significant changes in cTnT of the patients undergoing ECT (Fig 1). The cardiac TnT concentration was 0.008±0.006 ng/ml before the 1^st^ ECT procedure and 0.008±0.004 ng/ml after the 9^th^ procedure. The ANOVA did not show any significant differences for any of the consecutive cTnT measurements (*p = 0*.*36*). In our study, there was no significant correlation between cTnT and spatial QRS-TA (*p = 0*.*47*).

### The correlation of comorbidities and medications with ECG parameters and cTnT concentration

There was no significant differences in ECG parameters and cTnT concentration depending on gender, age, comorbidities (diabetes, arterial hypertension) or major psychiatric diagnosis. Moreover, psychotropic drugs had no impact on cTnT and ECG parameters. Due to the rather small size of the study group, the analysis of drug doses–ECG changes was not possible.

## Discussion

Although ECT has been used in psychiatry for several decades, its safety has not been explicitly demonstrated [5–8, 19, 20].

ECG parameters and cTnT are often used as surrogates in scientific research to indicate the risk of cardiovascular complications [21]. ECG is widely available, easy to perform and cost effective method and therefore it is valuable in screening and stratification of the adverse cardiovascular events risk. The available literature reports studies regarding the effect of ECT on ECG; these usually are single-centre trials, conducted on relatively small groups of patients, and focused on ECG evaluation before and after a single ECT procedure. In contrast, our study analyzed ECG recordings during the entire therapeutic cycle, with additional synchronous cTnT measurements. To the best of our knowledge, this is the first study regarding the spatial QRS-TA in patients treated with ECT.

In the available reports concerning ECT, various ECG parameters have been analyzed, the most common being the QT interval, the corrected QT interval (QTc) and its dispersion. It should be emphasized that the literature findings are inconsistent [22–26]. Masdrakis et al. [23] published data of 9 patients treated with ECT, with no prolonged QTc in ECG or adverse cardiovascular incidents. A recent study by Coskun et al. [27] showed no increase in ventricular repolarization heterogeneity (Tp-Te, Tp-Te/TQc, JTc and JTd) in patients treated with ECT.

Rasmussen et al. [24], on a group of 46 patients, found prolonged QTc dispersion immediately after the procedure. No correlation between the increased QTc dispersion and the occurrence of single ventricular and supraventricular premature beats was found. Moreover, the clinical usefulness of QT/QTc dispersion has been recently questioned [28–30]. The measurement itself is difficult due to frequent problems with the T wave end determination. Furthermore, the parameter was characterized as hardly repeatable with significant measurement errors [31].

The spatial QRS-TA is known to predict cardiovascular death, death from any cause, and cardiovascular incidents not resulting in death. Moreover, the spatial QRS-TA is a better mortality predictor than conventional risk factors, such as diabetes mellitus, arterial hypertension, myocardial infection, tobacco smoking and classic ECG parameters (including indices of left ventricular hypertrophy, ST-T segment abnormalities and left bundle branch block) [11, 32]. Numerous studies have demonstrated a strong correlation between the spatial QRS-TA and the risk of sudden cardiac death as well as ventricular arrhythmias [12, 33]. Spatial QRST angle values up to 50 degrees are considered to be a normal range [12]. Our main finding in the present study is the absence of any significant increase in spatial QRS-TA observed during the entire ECT course.

In our study, no effect of ECT on ST-T changes to ECG was observed. Only one patient was found to have ST segment depression on baseline ECG, and throughout the ECT cycle. Comparable results were published by Rumi et al. [34]; the authors treated 47 patients with ECT and found no significant ST-T changes or cardiac arrhythmias (ST segment depression was present prior the treatment and maintained during ECT in three female patients).

According to Martinez et al. [4], the values of troponin T were elevated among 11.5% of patients treated with ECT (yet in half of tested group the troponin concentration was elevated before the treatment, and in the remaining individuals it developed during ECT). Two patients with increased troponin concentrations died. It is well known that an increased concentration of cardiac troponin is a negative prognostic factor, irrespective of the cause of myocardial damage. Our findings did not demonstrate any significant increases in troponin T concentrations during ECT.

Differences in the results of the cited studies might be due to several factors, such as the heterogeneity of studied population, underlying cardiovascular diseases, the combined administration of psychopharmacotherapy, and finally a different anesthetic protocol.

Mutual correlations between troponin levels and QRST-A are complex sincerepresent different dimensions of cardiomyocytes damage and can be associated with different pathomechanisms. In our study, there was no significant correlation between these two parameters observed. The available data regarding the correlation of these parameters are inconsistent. In patients with sleep-related disorders, troponin T did not correlate with QRST-A [21], while in other studies conducted on high-risk patient groups, such as end-stage renal disease, this association was robust [16]. It seems that such a correlation can be modulated by different cardiac conditions, especially underlying coronary artery disease or heart failure.

Assessment of QRS-TA could be a potential marker of cardiac risk in patients undergoing ECT. QRS-TA could be used in combination with cardiac troponin test for stratification of patients at high risk of cardiovascular adverse events. Due to its reproducibility, accessibility and cost effectiveness it might be used in psychiatric care setting. However, further prospective research is needed to confirm utility of QRS-TA in ECT safety management.

### Limitations of the study

Although we believe that our results are of high value, there are limitations of the trial that need to be addressed. First of all, our study was a single-centre investigation. Included patients were relatively young without diagnosis of cardiovascular diseases. Thence, our results should not be extrapolated onto the group with high cardiovascular risk. Secondly, we performed one-time ECG evaluation one hour after ECT, that might not be enough to evaluate the risk of arrhythmia. Previous findings [24, 26] provided evidence of immediate cardiac electrophysiological disturbances. Therefore, continuous and long-term ECG monitoring might be helpful in elucidating the temporal correlation between ECT and arrhythmia.

Thirdly, although the study patients underwent physical examinations before ECT; echocardiography or exercise testing were not performed, what could have resulted in an underestimation of the cardiovascular events incidence. At last, one protocol of anesthesia (propofol and suxamethonium) was used in the study group. The protocols for anesthesia based on other drugs should be assessed separately.

## Conclusions

Our findings suggest no negative effects of ECT on the risk of adverse cardiovascular events, based on the spatial QRS-T angle and cardiac troponin T concentration. However, it is essential to continue studies to evaluate the long-term safety of ECT, as well as the risk of complications in subgroups of patients with baseline cardiovascular diseases, in order to identify the groups of patients at a particular risk of cardiac complications.
